# Physico-mechanical properties and bacterial adhesion of resin composite CAD/CAM blocks: An *in-vitro* study

**DOI:** 10.4317/jced.59548

**Published:** 2022-05-01

**Authors:** Mohamed M. Mokhtar, Dina S. Farahat, Waleed Eldars, Manal F. Osman

**Affiliations:** 1 Research Assistant, Department of Dental Biomaterials, Faculty of Dentistry, Mansoura University, Mansoura, Egypt; 2 Assistant Professor, Department of Dental Biomaterials, Faculty of Dentistry, Mansoura University, Mansoura, Egypt; 3 Associate Professor of Microbiology, Faculty of Medicine, Mansoura University, Mansoura, Egypt; 4 Professor, Department of Dental Biomaterials, Faculty of Dentistry, Mansoura University, Mansoura, Egypt

## Abstract

**Background:**

The recent introduction of CAD/CAM technology has been strongly impacting the workflow in dental clinics and labs. Among the used CAD/CAM materials, resin composite CAD/CAM blocks offer several advantages. The aim of this study was to evaluate the physico-mechanical properties and bacterial adhesion of a recently introduced nanoceramic hybrid material (Grandio Blocs) comparing it to a nanoceramic CAD/CAM material (Lava Ultimate).

**Material and Methods:**

A total of 82 specimens were prepared; 41 specimens from each material. For flexural strength testing, bar shaped specimens were sectioned from each material and flexural strength was evaluated using a three point bending test. For surface hardness, specimens with 2 mm thickness were prepared, polished and tested using Vickers micro-hardness tester. For wear evaluation, specimens were tested in a block on ring tribometer and the amount of weight loss was determined. A stylus profilometer was used to evaluate the surface roughness of disc shaped specimens in three directions. For the bacterial adhesion, the same specimens from the roughness test were used to evaluate the adhesion of Streptococcus mutans to the surface of each material after incubation for 24 hours. The correlation between surface roughness and bacterial adhesion was also investigated.

**Results:**

The nano-ceramic hybrid CAD/CAM material exhibited significantly higher flexural strength and surface hardness than the nano-ceramic CAD/CAM material. It also showed significantly lower surface roughness and surface bacterial adhesion and lower wear that was not significantly different. A positive correlation was found between surface roughness and bacterial adhesion of both materials.

**Conclusions:**

The nano-ceramic hybrid CAD/CAM material showed better physico-mechanical properties compared to the nano-ceramic CAD/CAM material which could be attributed to the use of nanohybrid filler system and an enhanced resin matrix structure.

** Key words:**CAD/CAM blocks, nano-ceramic hybrid, flexural strength, wear, surface hardness, surface roughness, bacterial adhesion.

## Introduction

Computer aided design and computer aided manufacturing (CAD/CAM) is considered one of the important advances in dentistry that has been causing a significant impact on the dental laboratory workflow in the last decade. Initially, the use of this technology was limited to glassy ceramics (1), however, its use has been extended to the processing of different materials such as polycrystalline ceramics, different metal alloys and composite and acrylic resins. This could be attributed to introduction of new digitalization technologies and tools and the efforts directed towards modifying several materials to adjust to the milling procedures (2). CAD/CAM technology provides standardized manufacturing, quality control and cost, labor and working time reduction (3). For the production of esthetic restorations using CAD/CAM technology, two main types of materials are commonly used; ceramics and resin based composites. Dental ceramics possess superior esthetics and biocompatibility among restorative materials (4). In addition, they exhibit good mechanical properties with a relatively high elastic modulus that affects their stiffness. Nevertheless, brittleness of dental ceramics is one of their major drawbacks that can result in catastrophic failure and affect their longevity during service. Moreover, these materials are highly susceptible to chipping or cracking during the milling procedure (5) and their hardness can result in the abrasion and wear of opposing natural teeth (6).

Recently, the use of CAD/CAM technology to fabricate resin composite restorations has been strongly advocated. However, the use of resin composites in the fabrication of indirect laboratory restorations began in the 1980s. The aim was to increase the degree of monomer conversion which led to the enhancement of the material’s biocompatibility and mechanical and physical properties and to reduce polymerization shrinkage and stresses that can adversely affect bonding to the tooth structure (7). Nonetheless, these materials still exhibited the intrinsic drawbacks due to the use of hand building techniques, such as pores and inhomogeneities (8). The industrial processes used in the manufacturing of resin based CAD/CAM blocks were reported to improve material homogeneity, decrease pores and flaws and increase material reliability (9). The polymerization process is carried out using ultra high temperature and high pressure (>150 MPa) in high-performance standardized industrial settings, rather than the traditional heat and photo-polymerization, producing highly polymerized materials. The manufacturing process also allows for the augmentation of the filler volume fraction which might not be possible with traditional composites (2).

CAD/CAM resin composites display lower hardness when compared to ceramics resulting in decreased abrasion of opposing natural teeth in addition to lower incidence of wear of milling tools making it a more favorable candidate for the machining process (10). Likewise, resin composite CAD/CAM materials exhibit lower brittleness making it less liable to catastrophic failure during service or chipping during manufacturing. Other advantages of CAD/CAM composite materials include better machinability, faster processing, lower cost, an elastic modulus similar to that of dentin and stronger bonds to resin cements due to their comparable structures (11). According to their microstructure, CAD/CAM resin composite materials are generally classified into polymer infiltrated ceramic network (PICN) materials or materials composed of resin matrix with dispersed fillers. PICN materials are true hybrid materials in which a porous ceramic network is infiltrated by resin polymer (12). On the other hand, resin matrix with dispersed fillers materials consist of highly cross-linked resin matrices with an increased volume fraction of filler particles. These materials are based on the traditional filler-resin mixing composite technology while making use of innovations in resin monomer compositions, initiation systems, curing modes and filler loading (11).

The success, longevity and reliability of a dental restoration during service relies greatly on the properties of materials used in its manufacturing. So, the aim of this study was to report on some of the mechanical properties (flexural strength, surface hardness and wear), physical properties (roughness) and bacterial adhesion of a recently introduced CAD/CAM resin composite block and compare it to a commercially available composite block material. The null hypothesis was that there will be no difference between the properties of the two materials.

## Material and Methods

The composition, manufacturer, batch no and shade of materials used in this study are shown in Table 1.

**Table 1:**
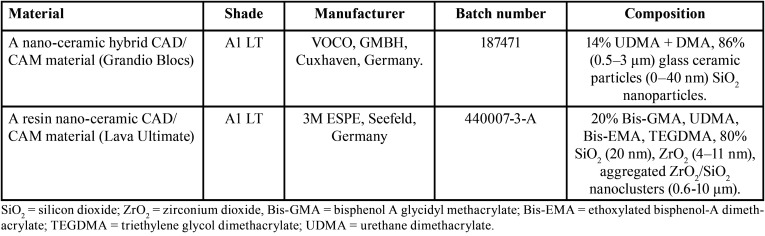
Composition, manufacturer, batch no and shade of materials used in this study.

-Sample size calculation

Sample size calculation was based on mean differences of flexural strength between studied groups retrieved from previous study by Lawson *et al*. (10) with a design similar to the present study. Using G*power (version 3.0.10) to calculate sample size based on 2-tailed test, α error =0.05 and power = 90.0% and effect size =2.01, the total calculated sample size was determined to be 10 in each group.

-Preparation of specimens

A total of 82 specimens (41 from each material) were sectioned from the CAD/CAM blocks using a water-cooled low speed diamond wafering blade mounted on a precision saw (Isomet 4000, Buehler, Lake Bluff, Illinois, USA). Dimensions of the specimens varied according to the type of intended testing. The specimens then were polished according to manufacturers’ instructions.

-Flexural strength test:

Flexural strength was measured using a three point bending test in a universal testing machine (Instron, Model 3345; Norwood, MA, USA). Twenty bar shaped specimens (ten from each material) with the dimensions (14 x 4 x 2 mm) were mounted on vertical supports with a 10mm span. A vertical compressive load was applied to the center of each specimen at a crosshead speed of 1 mm/min till fracture. The maximum load at fracture was recorded and flexural strength (σ) was determined in MPa according the following formula σ = 3FL / 2wd2

Where F is the maximum load at fracture, L is the span between vertical supports, w is the width of the sample and d is the depth of the specimen (13).

-Hardness test:

Surface hardness was determined using a micro-hardness tester with a Vickers diamond indenter (Jinan Precision Testing Equipment CO, Model HV-1000ltd, China). Ten specimens from each material were sectioned with a thickness of 2 mm. For each specimen, three indentations were made each being no closer than 0.5 mm with an applied load of 50 g for 15 s dwell time. The Vickers hardness values were calculated for each material.

-Wear test:

Twenty specimens, 10 from each material, were sectioned with dimensions of 10 x 7 x 6 mm, and polished according to manufacturers’ instructions. A block on ring tribometer was used to measure the wear of the tested specimens. First, each specimen’s weight was determined using a digital balance, and then it was fixed in the tribometer against a stainless steel ring (Antagonist). The hardness of the ring was 63 HRC and its thickness was 74 mm. The test was performed under an applied load of 0.7 bar with a sliding speed of 65 rpm and continued for 15 minutes. The weight of each specimen was determined again using the same balance and the wear value was determined for each specimen by calculating the amount of the weight loss, according to the following equation:

Wear = Initial weight - final weight.

-Surface roughness:

Twenty disc shaped specimens (10 from each material) with 2mm thickness and 8mm diameter, were cut from the blocks then polished following the manufacturers’ instructions. A stylus contact profilometer (Surftest Sj-210, Mitutoyo, Corp, Kawasaki, Japan) was used to evaluate the surface roughness of each specimen. Three measurements were carried out in different areas with a tracing length of 0.25 mm, a scanning speed of 0.5 mm/s and a resolution of 0.01 μm. The average roughness, Ra, was calculated for each material.

-Bacterial adhesion.

To evaluate the bacterial adhesion to the surface of each of the materials under investigation, the same specimens from the roughness test were used in addition to an extra specimen from each material for SEM investigation. Clinically isolated *Streptococcus mutans* were aerobically grown in brain heart infusion broth for 24 h at 37oC. Then, 22 tubes were filled with 1ml of the bacterial suspension with an adjusted turbidity of 0.5 McFarland turbidity standard. Each specimen was inserted in one tube using a sterile tool and incubated for 24 h at 37oC. Twenty specimens were washed with saline to remove non-adherent bacteria, then each specimen was inoculated in 1ml saline and mixed in a vortex for 3 minutes. One microliter was taken from each of the resultant suspensions, striated in blood agar plates and incubated for 24 h at 37o C. Then, colony forming units (CFUs) were counted, and CFU/mL was calculated.

To assess bacterial adhesion to the materials using scanning electron microscopy, two specimens (one from each material) were rinsed with saline to remove non-adherent bacteria then fixed in 10% formaldehyde for 1 h. Dehydration was performed using increasing concentrations of ethanol and finally the specimens were dried in a bacteriological incubator at 37oC for 24 hours. Specimens then were gold sputtered and bacterial adhesion was investigated using a SEM (JEOL- JSM- 6510LV- Japan) at an accelerated voltage of 30 kv and a magnification of 5000X.

-Statistical analysis:

Data was collected and analyzed using SPSS software for Windows version 20.0 (IBM SPSS, SPSS Inc., Chicago, IL, USA). Data were presented as the means ± standard deviation (SD) for parametric data after testing normality using Shapiro-Wilk test. The statistical significance of differences was evaluated by the Student’s t-test analysis. Pearson correlation coefficient was calculated to determine the linear inter-relationship between the surface roughness and bacterial adhesion for the investigated materials. Differences were considered significant for *p < .05.

## Results

Table 2 shows the means and standard deviations of flexural strength, Vickers hardness values, wear values, surface roughness and bacterial adhesion of Grandio Blocs and Lava Ultimate. The results of the student’s t-test showed that the flexural strength and surface hardness of Grandio Blocs were significantly higher than Lava Ultimate (*p* < 0.001) whereas the there was no significant difference in wear values of both materials (*p*=0.093). On the other hand, results of the student’s t-test revealed that Grandio Blocs exhibited significantly lower surface roughness and bacterial adhesion values (*p* < 0.001) compared to Lava Ultimate. The calculated Pearson’s correlation coefficient showed that there was a significant positive correlation between surface roughness and bacterial adhesion of both the materials (Grandio Blocs r=0.68 *p*=0.03, Lava Ultimate r=0.81 *p*=0.004).

**Table 2:**
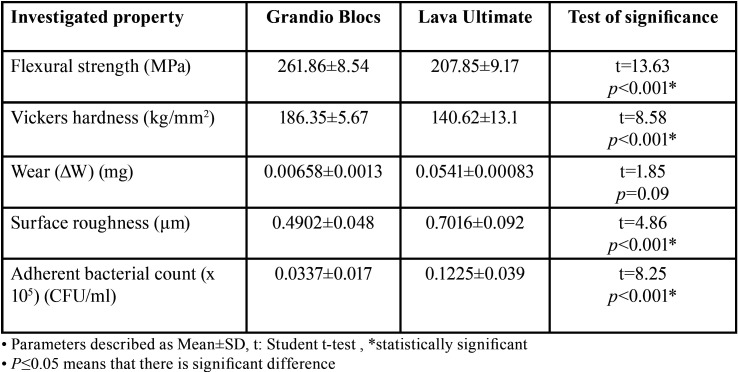
Comparison of flexural strength, Vickers hardness, wear, surface roughness and bacterial adhesion of Grandio Blocs and Lava Ultimate CAD/CAM blocks.

**Figure 1:**
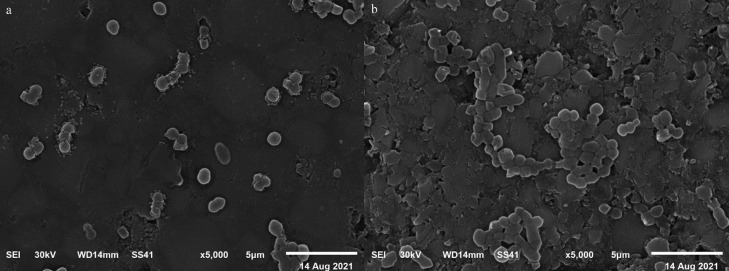
Scanning electron photomicrographs of *S. mutans* adherent to the surface of a. Grandio Blocs, b. Lava Ultimate.

Figures 1 a and b show scanning electron photomicrographs of *S. mutans* adherent to the surface of Grandio Blocs and Lava Ultimate respectively. With Grandio Blocs, the number of adhering cocci was relatively lower than Lava Ultimate. The cocci are found in pairs or smaller clusters on the surface of Grandio Blocs, while they are found in larger clusters on the surface of Lava Ultimate.

## Discussion

In the present study properties of a recent nano-ceramic hybrid CAD/CAM material (Grandio Blocs) and a nanoceramic CAD/CAM material (Lava Ultimate) were evaluated for flexural strength, surface micro-hardness, wear, surface roughness and bacterial adhesion. Grandio Blocs showed significantly higher flexural strength, microhardness, non-significantly different wear and significantly lower surface roughness and bacterial adhesion when compared to Lava Ultimate so the null hypothesis was rejected.

Flexural strength is an indication of the resistance of a material to fracture under bending forces. A three-point flexural test was selected in this study due to the less complex distribution of stresses when compared to other methods as biaxial flexural strength test (14). It was carried out following ISO 6872 international standard for flexural testing, modified to solve the difficulty in obtaining the specified specimen dimensions from commercially available CAD/CAM blocks (15). The flexural strength of Grandio Blocs was significantly higher than Lava Ultimate. This could be attributed to the higher filler loading in Grandio Blocs (86 wt%) compared to Lava Ultimate with a filler loading of 80 wt%. Grandio Blocs is a nano-ceramic hybrid material which contains a mixture of ceramic nanosized particles and conventional filler particles. The use of different filler sizes enabled the loading of higher amounts of strong filler particles that could prevent crack propagation and hence increase the material’s strength. Kim *et al*. and Rastelli *et al*. reported the intimate correlation between increasing filler load and the increased flexural strength, which is a finding of this study (16,17). Additionally, Lava Ultimate is a nano-filled composite block that contains zirconia and silica nanomers (4-20 nm) and nanoclusters (0.6-10 um). The conFiguration of a nanocluster comprises internal porosity infiltrated by a second phase and would be predicted to be inherently weaker than a similar dense silica particle and hence the lower strength of the nano-filled material. However, such nanoclusters were reported to be highly effective reinforcing agents in a polymeric matrix in a study by Randolph *et al*. (18). On the other hand, the resin matrix composition of the CAD/CAM blocks might also be an important factor affecting their mechanical properties. Generally, Urethane dimethacrylate (UDMA), which is the main monomer in Grandio Blocs, exhibits higher mechanical properties compared to Bis-GMA, Bis-EMA and TEGDMA. Lava Ultimate, on the other hand, contains all these monomers which may explain the lower mechanical properties compared to Grandio Blocs (19).

Hardness of a material indicates its surface resistance to indentation and the subsequent ease of finishing and polishing and resistance to scratching during service. Vicker’s microhardness test was selected in this study since it is suiTable for testing several types of materials especially brittle ones. The nano-ceramic hybrid material (Grandio Blocs) showed significantly higher hardness values than Lava Ultimate. It could be assumed that increasing the filler load in Grandio Blocs increased its surface hardness when compared to Lava Ultimate since the hardness of fillers is approximately ten times as much as the resin (20). The effect of filler loading on surface hardness seen in this study has been also reported in several previous studies (21,22). The higher resin concentration and composition in Lava Ultimate also plays an important role in its lower surface hardness. The weaker resin matrix may be chipped away and cause the dislocation nano-ceramic clusters from the surface during polishing leaving a porous and non-homogenous surface, where the Vickers indenter can penetrate easily into exposed resin during the testing procedure.

Wear can be defined as the gradual loss of surface material between two contacting bodies that are in relative motion under a given load (23). Wear properties of a dental material are important as they can affect its clinical service and occlusal contacts overtime. In this study, a two-body wear test was used. It resembles the direct friction between the restoration material and tooth and was selected because it is practical, economical, and commonly used. Grandio Blocs showed non-significantly lower wear values than Lava Ultimate. The nanofiller technology used with Lava Ultimate comprises nanoclusters formed from loosely bonded aggregates of nanoparticles which can break away during polishing and wear in attempt to help gloss retention and decrease wear. However, Lava Ultimate showed non significantly higher wear values in this study. As the surface resin is removed due to contact with an opposing surface, nanofiller particles separate from the nanoclusters. These loose nanoparticles become lodged between the two surfaces and could exert an abrasive effect on the surface of the material significantly increasing its wear (24). In addition, the Lava Ultimate has a higher concentration of surface resin that can be easily abraded compared to Grandio Blocs.

Surface roughness is a critical parameter in selecting a restorative material, as it is necessary for color stability, surface gloss retention and decreased retention of micro-organisms (25). A Stylus contact profilometer was used to assess the surface roughness parameters for both materials used in this study after polishing the surfaces of all specimens according to their manufacturer’s recommendations. Grandio Blocs showed significantly lower roughness when compared to Lava Ultimate. The nano-ceramic material comprised higher resin content than the hybrid one and the polymer-rich surface layer is often clinically removed by finishing and polishing and could be easily worn out. As a result roughness on the polished surface to varying degrees may occur depending on the polishing system and material used. Consequently, the densely packed nano-filler clusters might be displaced from surface during polishing forming intermediate slurry that causes a three-body wear leading to rougher surfaces (24).

Bacterial adhesion and biofilm formation significantly affect the esthetic properties and longevity of a restoration. In this study, *S. mutans* was selected as it is one of the most studied in oral microbiology and is contemplated as the main etiological factor in dental caries formation (26). The results of the current study show that Grandio Blocs showed significantly lower bacterial adhesion to the surface of its specimens compared to Lava Ultimate. Bacterial adhesion is significantly affected by several factors among the most important are the surface roughness, and material composition. Generally, bacterial adhesion increases with an increase in surface roughness due to the difficulty in cleaning, increase in contact area, and the shielding of the microbial cells from shear forces (27). In this study, the calculated Pearson’s correlation coefficient suggested that there is a significant positive correlation between surface roughness and bacterial adhesion in both materials. Furthermore, the SEM images recorded in this study for the surface of Grandio material revealed a lesser number of adherent bacteria with a smoother surface compared to a rougher surface of Lava Ultimate with a higher number of adhering bacterial cells. A positive correlation between surface roughness and bacterial adhesion, as the one seen in this study, has been also previously reported in several studies (28–30). Moreover, Ionescu *et al*., reported a positive correlation between the amount of resin matrix on the surface and biofilm formation and a negative correlation between the amount of inorganic filler and biofilm formation (31). Some resin monomers as TEGDMA can increase bacterial growth whereas UDMA and Bis-GMA promote glycosyltransferase activity of cariogenic bacteria (32,33). Also, salivary and bacterial enzymes can biodegrade the resin matrix (34). So, it could be speculated that Lava Ultimate would show higher surface bacterial adhesion. Nonetheless, it was suggested that the pre-polymerization process in CAD/CAM resin blocks can help decrease the uncured resin matrix components and thus decrease bacterial adhesion and biofilm formation (35).

## Conclusions

The use of nanohybrid filler technology and modified resin matrix composition in the nano-ceramic hybrid resin composite CAD/CAM blocks (Grandio Blocs) improved most of its physico-mechanical properties and reduced bacterial adhesion compared to the nano-ceramic CAD/CAM material (Lava Ultimate).
